# Curcumin, Coenzyme-Q10, and Bioactive Compounds in Ashwagandha Extract: Multi-Targeting Potential of Co-Administered Natural Health Compounds as Therapeutic and Preventative Interventions in Alzheimer’s and Parkinson’s Disease Models

**DOI:** 10.3390/nu18121986

**Published:** 2026-06-19

**Authors:** Keanna Dube, Alex Stoinescu, Siyaram Pandey

**Affiliations:** 1 Department of Integrative Biology, University of Windsor, 401 Sunset Avenue, Windsor, ON N9B 3P4, Canada; dube112@uwindsor.ca; 2 Department of Chemistry & Biochemistry, University of Windsor, 401 Sunset Avenue, Windsor, ON N9B 3P4, Canada; stoines@uwindsor.ca

**Keywords:** Alzheimer’s disease, Parkinson’s disease, natural health products, autophagy, neuroinflammation, oxidative stress, ashwagandha, coenzyme-Q10, curcumin, nanoformulations

## Abstract

Background/Objectives: Neurodegenerative diseases such as Alzheimer’s disease (AD) and Parkinson’s disease (PD) represent a growing public health concern. Both disorders are driven by mitochondrial dysfunction, oxidative stress, impaired autophagy, neuroinflammation, and neuronal loss. Single-target therapeutics have failed to halt disease progression, highlighting the need for multi-target interventions that address the complex and interconnected nature of neurodegeneration. Natural health products (NHPs) such as curcumin (CUR), coenzyme-Q10 (CoQ10), and Ashwagandha (ASH) possess antioxidant, anti-inflammatory, neuroprotective, and neurotrophic properties that may collectively address this complex pathology. However, poor bioavailability and hydrophobicity have limited clinical translations. Novel formulations, including nanomicellar Ubisol-Q10 (UQ) and water-solubilized ASH (PTS-ASH), have demonstrated enhanced metabolic uptake and neuroprotective efficacy in preclinical models. Moreover, co-administered NHPs, such as CUR + CoQ10 and CoQ10 + ASH, may provide further benefits by diversified targeting of disease pathways. Methods: This review presents an integrative interpretation of a combined UQ + ASH “tonic” in transgenic AD and paraquat-induced PD animal models using previously published qualitative immunohistochemical and functional results. This report constructs a proposed mechanistic model illustrating how these compounds may interact across multiple stages of disease AD and PD progression. Results: Based on comprehensive interpretation of the previous published reports, consistent trends suggest UQ stabilizes mitochondrial energetics and suppresses oxidative damage upstream, whereas ASH promotes downstream repair and synaptic modulation. Combined administration remained as providing balanced neuroprotective and functional outcomes. Conclusions: These interpretations of published reports and proposed mechanistic models aim to improve the translation and support the therapeutic potential of multi-component natural interventions for neurodegenerative diseases and highlight the importance of bioavailability-enhancing formulations in future preclinical and clinical research.

## 1. Neurodegenerative Disease Prevalence and Shared Mechanisms

Neurons are long-lived post-mitotic cells that cannot divide to dilute cellular waste, making autophagy and mitophagy particularly important processes [1]. The brain consumes 70% of the body’s glucose, requiring tremendous amounts of adenosine triphosphate (ATP) [2]. Through the aging process, neuronal function and ATP production decrease leading to a high vulnerability to oxidative stress. As the primary energy source in neurons, mitochondria play a critical role in supporting synaptic plasticity, synaptic transmission, redox signaling, and overall neuronal survival [1]. As mitochondrial function becomes altered through disease and aging, they fail to provide the necessary ATP for vital processes like synaptic transmission and the maintenance of ionic gradients [3]. Although the body maintains mitochondrial health through antioxidant defenses and mitophagy, impairment of these systems leads to rapid neuronal malfunction, declines in cognition, and reduced quality of life.

Neurodegenerative disorders such as Alzheimer’s disease (AD) and Parkinson’s disease (PD) are characterized by complex, multifactorial pathologies involving oxidative stress, mitochondrial dysfunction, impaired autophagy and proteostasis, neuroinflammation, and progressive synaptic and neuronal loss [4]. Although AD and PD present with distinct impairments, they exhibit remarkably similar core pathological features, where mitochondrial dysfunction and abnormal protein aggregation drive neuroinflammation [4,5,6]. Despite extensive efforts, therapeutic strategies targeting just one or two of the various disease pathways have been unsuccessful in halting disease progression [6,7,8]. Therefore, a therapeutic regimen which addresses multiple of the disease pathways simultaneously and is well-tolerated over long periods of intake may provide better outcomes in neurodegenerative disease treatment.

Natural health products (NHPs) represent a broad group of health-related substances derived from a variety of naturally occurring sources [9]. NHPs have been used for human consumption for thousands of years across a variety of different cultures as a method of therapeutic intervention. They can be well-tolerated over a long period of time, unlike many chemically derived pharmaceutical options. NHPs exhibit neuroprotective, antioxidant, and anti-inflammatory properties that may help manage neurodegeneration and slow its progression, offering a less invasive alternative with fewer side effects [9].

### 1.1. Pathology and Development of AD

In Canada, an estimated 77,000 Canadians are living with diagnosed dementia, with this number expected to double by 2050 [10]. This rapid increase in the number of Canadians living with dementia is placing an unsustainable burden on both formal and informal healthcare systems [11]. AD is driven by a multifactorial network of biochemical disturbances, including amyloid-β (Aβ) accumulation, tau hyperphosphorylation, impaired autophagy, neuroinflammation, oxidative stress, and synaptic dysfunction [12]. Extracellular Aβ aggregates disrupt neuronal communication by interfering with synaptic signaling, particularly at the postsynaptic membrane, and forming β-sheet structures that impair receptor function and ion homeostasis [7]. Aβ buildup promotes downstream tau pathology, where tau becomes hyperphosphorylated, detaches from microtubules, and aggregates into neurofibrillary tangles that disrupt axonal transport and synaptic integrity [13,14,15,16]. Simultaneously, impairment of autophagy limits the clearance of misfolded proteins and damaged organelles, exacerbating both Aβ and tau accumulation, while promoting mitochondrial dysfunction and lysosomal failure [17,18]. These processes trigger chronic neuroinflammation, in which sustained activation of microglia and astrocytes leads to the release of pro-inflammatory cytokines and reactive oxygen species (ROS), creating a toxic environment that accelerates neuronal injury [16,19]. Oxidative stress, driven by mitochondrial dysfunction and excessive ROS production, damages lipids, proteins, and DNA, further impairing neuronal survival. Together, these interconnected mechanisms result in synaptic loss and network dysfunction, which closely correlate with the brain atrophy and progressive cognitive decline observed in AD.

### 1.2. Pathology and Development of PD

PD arises from a complex interplay of environmental exposures, internal biological processes, and genetic susceptibility [20]. Roughly 95% of PD cases are sporadic and appear after the age of 60. Genetic factors such as both dominant and recessive autosomal genes account for approximately 5–10% of PD patients [21]. Mutations in SNCA, LRRK2, Parkin, PINK1, and DJ-1 are commonly linked to both familial and sporadic Parkinson’s disease, with mutations in the latter three genes typically associated with early-onset forms of the disease [21]. Environmental factors such as exposure to pesticides, head trauma, and lifestyle factors have also been linked to increasing the biological pathology of PD [20,21]. The primary pathological characteristic of PD is the progressive loss of dopaminergic (DA) neurons in the substantia nigra pars compacta [22]. These neurons connect to the striatum to facilitate movement and coordination. DA neurons in the substantia nigra are particularly vulnerable to neurodegeneration due to high metabolic demands during dopamine metabolism and subsequent vulnerability to oxidative stress [23]. Therefore, their depletion causes severe reduction in dopamine levels and subsequent decline in motor abilities, such as tremor, rigidity, and bradykinesia [22]. However, there is a symptomatic delay where roughly 30% of DA neurons are already lost before symptoms become apparent [21]. Moreover, despite the substantia nigra being the primary target, PD pathology may initiate in the olfactory bulb or gut before spreading in a rostrocaudal manner through the brain [22]. On a more microscopic level, PD involves the buildup of cytoplasmic protein aggregates called Lewy bodies and Lewy neurites composed primarily of α-synuclein, but also ubiquitin, tau, and other cytoskeletal proteins [24,25]. α-synuclein misfold into β-sheet-rich filamentous structures which have prion-like properties and can spread their misfolding nature between neurons [24]. Like AD, the toxicity of these soluble oligomers and protein deposits cause a cascade of dysfunctional mitochondria, oxidative stress, impaired autophagy, neuroinflammation, and exacerbated neuronal malfunction [22].

## 2. Current Pharmaceutical Therapeutic Interventions

Current treatments of neurodegenerative disorders aim to alleviate downstream consequences by targeting the clearance of pathological proteins and regulating neurotransmitters related to the disease. In AD treatment options, there are no disease-modifying drugs to effectively target all five etiologies simultaneously [26]. Seven drugs approved by the Food and Drug Administration (FDA) are currently prescribed to patients. These include cholinesterase inhibitors (Donepezil, Rivastigmine, and Galantamine), which focus on enhancing the amounts of cholinergic transmission across the synaptic cleft. Similarly, *N*-methyl-*D*-aspartate (NMDA) receptor antagonists, such as Memantine, work by binding to NMDA receptors, preventing excessive calcium influx and protecting neurons from the excitotoxicity caused by glutamate dysregulation in AD [4]. The combined therapy of these treatments has been explored in severe AD cases. The newest approved treatment of AD is anti-amyloid monoclonal antibodies (Lecanemab and Donanemab), which target the removal of Aβ plaques [27]. These lab-engineered antibodies bind to specific forms of Aβ, marking them for clearance by the immune system. However, the treatments may lead to adverse effects, such as edema and hemorrhage [7,8,28].

In PD, current treatment options focus on improving quality of life and managing symptoms. The gold standard treatment in PD is carbidopa–levodopa which works by replacing dopamine in the brain and preventing dopamine from reaching the periphery [29]. Catechol-O-methyltransferase (COMT) inhibitors work well in combination with levodopa by preventing its degradation [30]. Another treatment option is dopamine agonists which mimic dopamine by stimulating DA receptors in the brain [31]. Monoamine oxidase B (MAO-B) inhibitors work to increase dopamine levels in the brain by blocking the enzyme that breaks it down [29]. Surgical treatments for PD include deep brain stimulation (DBS), which modulates the electrical signals in the basal ganglia to restore normal motor circuit activity [32]. No standard treatment approved for PD targets the core pathological drivers such as mitochondrial dysfunction, oxidative stress, impaired autophagy, or protein aggregation.

## 3. Potential for Nutraceutical and Natural Extracts as a Therapeutic Intervention

Despite extensive efforts, therapeutic strategies targeting just one or two of the various disease pathways have been unsuccessful in halting disease progression of both AD and PD. There is a need for a multi-targeting intervention that address the complex pathological processes related to neurodegenerative disorders. Equally important, therapeutic agents used to treat chronic diseases should be tolerable over long periods of time without harmful side effects or toxicity.

### 3.1. Curcumin

Curcumin (diferuloylmethane; CUR) is a hydrophobic polyphenolic compound found in high amounts in turmeric (*Curcuma longa*) [9]. CUR has shown promise in its anti-amyloid effects by its preferential binding to Aβ-oligomers, fibrils, α-synuclein and phosphorylated tau to inhibit amyloid aggregation in AD and PD [33,34,35,36]. It also has shown antioxidative properties specific to AD and PD cellular dysfunction by scavenging and quenching harmful oxidative radicals, promoting antioxidant enzymes in the brain, and protecting cells against oxidative damage by increasing levels of glutathione (GSH), preventing mitochondrial permeabilization [37,38,39]. Another very important pathological aspect of AD and PD is chronic and prolonged neuroinflammation, which leads to accelerated protein aggregation and accumulation, synaptic dysfunction and neuronal death [40]. To this point, CUR has shown immuno-modulatory effects by inhibiting HMGB1-RAGE/TLR4-NF-κB pro-inflammatory signaling cascade [41] and blocking NLRP3 inflammasome activation [42] to ultimately alleviate chronic upstream and downstream neuroinflammation.

#### CUR Bioavailability and Methods of Enhancing Metabolic Uptake

Despite the disease-modifying evidence of CUR, its lipophilic properties, chemical instability, and rapid metabolism lead to very poor bioavailability and, therefore, effective doses of CUR are unrealistically high [43]. To address this issue, recent advances in alternative formulations have been explored. For example, co-administration with piperine from black pepper, has inhibitory effects on glucuronidation, which is the liver and intestinal process of CUR solubilization. Bypassing this process leads to rapid metabolism, therefore increasing the bioavailability of CUR by 20-fold [44]. Another method involves re-formulation as nanoparticles of different types, including nanoglobules and hydrogel nanoparticles [45,46]. These work by surrounding hydrophobic CUR in a hydrophilic shell leading to protection against hydrolysis and enzymatic degradation, ultimately slowing down the release of the active compounds over longer periods of time [47]. A noteworthy formulation called “Theracurmin” resulted in 40-fold higher bioavailability when administered orally to rats and translated to 27-fold increased bioavailability in humans when compared to standard CUR powder [48]. Another method to solving the bioavailability dilemma is manipulation of CUR’s delivery systems, such as incorporating lipid-based carrier molecules which solubilize the lipophilic molecules of CUR leading to faster absorption and higher peak plasma levels [47]. Nanoformulations of CUR have been explored preclinically to treat drug-resistant epilepsy showing strong potential in rodent models of epilepsy through enhanced bioavailability, improved brain delivery and seizure suppression [49].

### 3.2. Coenzyme Q10

Coenzyme Q10 (CoQ10) is an insoluble component and key player of the mitochondrial electron transport chain (ETC). CoQ10 has been widely shown to protect against oxidative damage and boost cellular energy (ATP), which in turn, preserves neuronal hardiness if subjected to conditions of metabolic stress [50,51]. It exists in two forms: ubiquinone, which is oxidized and acts as an electron acceptor to feed back into the ETC, and ubiquinol, which is the reduced form and acting as an electron donor and antioxidant [52]. Since AD and PD neurodegenerative processes parallel in mitochondrial destabilization and permeabilization, oxidative stress, and impaired redox cycling, CoQ10 contends as a suitable supplement for disease-modifying effects [53]. Preclinical animal studies of AD and PD models found that oral supplementation of CoQ10 resulted in reduced amyloid pathology, oxidative stress, neuronal loss, and improved behavioral and functional measures [54,55,56,57]. However, CoQ10 being lipophilic poses problems with poor bioavailability, and the effective doses used in preclinical studies were between 1200 and 2400 mg/kg/day. The translation of CoQ10 to clinical studies with humans is poor, resulting in low efficacy against neurodegenerative progression [5,58].

#### CoQ10 Bioavailability and Methods of Enhancing Metabolic Uptake

Clinical results using CoQ10 as a potential therapy for AD and PD, although unsuccessful, did confirm the safety of high doses of supplementation [5,58]. Therefore, the oil-soluble compound poses a similar challenge as CUR. Poor bioavailability and high effective doses do not translate well to humans, and therefore novel formulations are being explored. For example, Idebenone is a synthetic analog of CoQ10 which consists of a shorter and less lipophilic isoprenoid side chain, found to increase antioxidant capacities and rapid absorption in the intestinal tract [59,60]. Water-solubilized nanomicellar formulations of CoQ10, such as Ubisol-Q10 (UQ), have shown promise in preclinical animal models of AD and PD to slow or even halt degeneration at doses as low as 6 mg/kg/day [12,61,62,63,64]. UQ combines hydrophobic CoQ10 with polyoxyethany-α-tocopheryl sebacate (PTS), a synthetic amphiphile derived from vitamin E, in a 1:2 molar ratio. The amphiphilic nature of the compound forms a nanomicelle which allows the active component to bypass digestive processes, protecting CoQ10 from degradation in the stomach and leading to more effective transport and absorption in tissues, including the brain [61]. Notably, the protective effects of UQ can be attributed to stabilization of mitochondria by blocking BAX activity [65].

### 3.3. Ashwagandha (Withania somnifera)

*Withania somnifera* (WS), also commonly known as Ashwagandha (ASH), contains several bioactive compounds, each with their own beneficial actions. Withanolides are the primary active components which provide antioxidative properties through upregulation of enzymes such as SOD, GPX, and CAT, promote neurite outgrowth and synaptic remodeling, and enhance autophagic processes to rid abnormal proteins [66,67]. Withaferin A is one of the most potent withanolides, which inhibits NF-κB signaling to reduce neuroinflammation and activates Nrf2-related antioxidant signaling in response to cellular stress [68,69,70]. Flavonoids and other polyphenols are present in ASH in lower doses, but provide synergistic support such as ROS scavenging, supporting mitochondrial function, and having anti-inflammatory effects [71]. ASH has shown neuroprotective effects against AD- and PD-specific neurodegeneration primarily through regulation of neurotrophic signaling, suppression of inflammatory pathways, and promotion of synaptic plasticity and synaptic repair [72,73]. Similarly, the active compounds of ASH have been shown to restore Aβ-induced memory loss and promote axonal reconstruction [9]. Interestingly, withaferin A has been suggested to modulate oxidative stress specific to individual cellular context [74]. With respect to neurotransmission, ASH has been suggested to inhibit the activity of acetylcholinesterase, an enzyme heavily involved in learning and memory formation [75]. Early preclinical investigations of ASH in animal models of AD provide promising evidence of efficient Aβ clearance, and reversal of behavioral deficits with a dose of 1 g/kg/day [76]. Translating to humans, this dose is extremely high and may be impractical for every-day administration. Lower doses (100–300 mg/kg/day) have been tested in preclinical PD animal models and have shown potential to decrease oxidative stress, preserve DA neurons and reduce motor impairments [77,78]. A randomized, double-blind, placebo-controlled pilot study, which involved 50 subjects displaying mild cognitive impairment, were given 600 mg of ASH or a placebo over eight weeks. Results showed the treated group had significant improvements in immediate and general memory, executive function sustained attention, and information-processing speed compared to placebo-treated subjects [79]. Subjects from this study also demonstrated the safety of the potential therapy, with no side-effects reported. Controversy surrounding ASH has been reported from the Technical University of Denmark where the ban of the plant extract was initiated in 2020 due to reported harmful effects on sex hormones and other body systems, such as the liver. However, newer clinical evidence suggests that ASH root is generally safe and can improve fertility [80]. Regardless, this point emphasizes the necessity of implementing large-scale clinical trials on NHPs to confirm their safety and effective dosing strategies.

#### ASH Bioavailability and Methods of Enhancing Metabolic Uptake

Many active components within ASH, such as withanolides, are very hydrophobic and thus have poor bioavailability, making their clinical applications challenging. Human plasma concentrations of withanolides remain low after oral dosing, and only a small subset of active constituents are detectable in circulation [81]. The lipophilic steroidal lactones have limited aqueous solubility, resulting in low and highly variable systemic bioavailability following oral administration. Therefore, novel formulations, such as nanoformulations have shown potential to improve bioavailability, stability, and more targeted delivery of ASH [82]. Phytophospholipid nanocarriers are also being explored for their enhanced uptake of ASH components via encapsulation in phospholipid bilayers to improve stability, circulation time, and pharmacokinetics [83]. A micro-encapsulating formulation of 1.5% withanolides, called Zenroot™ (OmniActive Health Technologies Limited, Mumbai, India), underwent a small-scale clinical study with results suggesting a 2.1-fold increase in bioavailability at the very low dose of 125 mg, while also confirming safety and tolerability in healthy adults [84]. Moreover, water-soluble micellar formulations have also been investigated. For example, the PTS technology from UQ was extended to ASH (PTS-ASH), with a preclinical PD animal study showing its success at protecting against oxidative stress, neuroinflammation, DA neuronal loss, and motor function [63].

## 4. Combinatorial Effect of Co-Administered Natural Health Extracts

As is emphasized as a central perspective in this review, AD and PD are very complex diseases that involve multiple interwoven etiologies extending out from characteristic abnormal protein aggregation and accumulation. Therefore, therapeutics that focus on one or two of these pathways may not resolve the overall trajectory of disease progression. Perhaps for an intervention to be truly effective, it must be able to target the full cascade of neurodegeneration. Recent literature supports introduction of multi-modulatory treatments which target upstream stressors, intracellular control systems such as autophagy, and downstream neuronal and synaptic function [6,63,64,85,86]. NHPs have shown promise in their multi-targeting effects and may offer extended benefits when combined [44,61,63,64,87,88].

### 4.1. CUR + CoQ10

As mentioned previously, CUR shows positive results against AD and PD in its anti-amyloidogenic, neuroprotective antioxidative, and simultaneous upstream and downstream anti-inflammatory properties [33,34,35,36,37,38,39,40,41,42]. CoQ10 also has shown neuroprotective properties via mitochondrial support, boosting ATP, quenching ROS, and providing improved functional and cognitive outcomes in AD and PD preclinical studies [50,51,54,55,56,57]. Therefore, some studies have evaluated the dual benefits of both compounds combined, hypothesizing that they may yield more positive outcomes compared to independent administration. Indeed, one study by Kumar et al., 2023 [87], found that the combination therapy of 200 mg/kg CUR and 200 mg/kg CoQ10 orally administered to scopolamine hydrobromide-induced AD rats performed equivalent to Memantine-treated rats in memory and learning tests. Another study by Rasheed et al., 2025 [88], concluded that aluminum chloride-induced AD mice given 100 mg/kg of each CUR and CoQ10 had better cognitive performance in neuro-behavioral tests, restored redox balance, higher acetylcholine levels, and preserved neuronal vitality than untreated cohorts. Collectively, these studies support the therapeutic potential of CUR and CoQ10 co-administration as a multi-target strategy for neurodegenerative disease.

### 4.2. ASH + CoQ10

Previous reports from our research lab have investigated a combined “tonic” treatment of UQ, mentioned previously as a novel water-soluble formulation of CoQ10 with addition of amphiphilic PTS molecules, in combination with ASH, which has shown complementary effects and holds potential to combat disease progression of both AD and PD in preclinical animal models [61,63,64]. However, details of the mechanistic interactions between the active compounds in this combinatorial context have yet to be examined from the full body of evidence, and a more comprehensive perspective could be beneficial for further understanding of the complexity of combined formulations. Therefore, in the current report, we performed an integrative review of our previously published results featuring the combined administration of UQ + ASH [61,63,64].

## 5. Integrative Approach to Assessing Tonic Mechanisms on AD and PD

The data used for this review is already published but will be elaborated on and cross-assessed for important patterns and disease-specific mechanistic modulations from administration of UQ + ASH tonic. To do this, we will use the qualitative and semi-quantitative immunohistochemical outcomes from [61,63,64]. This integrated method of assessing two treatments in two pathologically complex disease models aims to provide a larger mechanistic understanding of the multi-target therapeutic strategies of UQ and ASH in neurodegenerative diseases. In addition, the current integrative review and proposed mechanistic model may be useful in investigation of other nutraceutical combinations with translational helpfulness to identify points of potential mechanistic interactions between active compounds, or even pharmaceutical agents.

### 5.1. Data Sources

Data were obtained from three independent experimental procedures involving a paraquat-induced PD rat model [61,63] and double-transgenic mouse model of AD [64]. All studies assessed multiple molecular, cellular, and functional markers via immunofluorescent assays following oral treatment with UQ and ASH, either administered independently or together in the tonic. All reported markers from each paper were included in the present analysis to maintain disease-specific biological context and to capture both shared and pathology-specific treatment responses.

### 5.2. How UQ + ASH Tonic Was Tested Using Preclinical Models of AD and PD

PQ is a well-known herbicide which induces acute mitochondrial redox cycling, continually generates ROS and aggressively injures affected neurons [89,90]. The double-transgenic AD model represents a slower and more exponential accumulation of amyloid pathology and metabolic stress [91,92]. Table 1 outlines the three studies using the tonic solution in AD and PD animal models and their methods of administration. Table 2 reports markers from each paper for disease-specific biological context and to capture both shared and pathology-specific treatment responses.

### 5.3. Comparative Analysis Strategy

Each published report provided marker expression values as a corrected total fluorescence (CTF) from three fields per animal per group using ImageJ 1.54 software [61,63,64]. For [63,64], CTF was converted to a percentage (%) of control group to give further context of marker results relative to their respective healthy saline-injected or wild-type control groups (control groups set to 100%) which allows relative comparisons across conditions. The only marker that was normalized to the unhealthy control group was Aβ, which was compared to untreated transgenic mice (set to 100%) since wild-type mice do not have the human transgenes, and therefore do not accumulate plaques.

Tonic treatment effects were evaluated across the following levels: independent effects of UQ and ASH given separately, combined effects of the tonic formulation, and inter-disease comparisons between PD and AD models. The analysis also incorporated important methodological differences including disease model severity (acute PQ vs. chronic transgenic pathology), euthanasia timing (4 months vs. 10 weeks post-PQ-injection endpoints in PD models), PTS carrier (vitamin E) concentration differences (0.1% vs. 20%), and ASH dosing (2 mg/mL in E-ASH and 1 mg/mL in PTS-ASH condition). Also of consideration is the healthy saline-injected groups receiving either the PTS control solution or regular water during the duration of the PD study [61]. These variables were included to evaluate their contribution to treatment-response variability.

It should be noted that the three studies included in the current analysis reported quantified values of fluorescent intensity as a secondary method of visualizing qualitative results, and therefore results can only be assessed for their general trends, directionality, and consistency. To corroborate our interpretation, we explored further mechanistically focused papers using CoQ10 and ASH separately [61,63,64]. No other results have been published using the combination tonic. We consider this review to be a starting point of general mechanistic targets based on compiled qualitative assessments and not a quantified statistical measure of absolute change.

### 5.4. Identification of Mechanistic Interactions

Mechanistic interactions between treatments are evaluated by interpreting patterns of inter-marker variabilities across cascade levels. Specifically, treatment effects are classified as either complementary, where UQ and ASH influenced distinct but interconnected pathways; convergent, where both treatments effected the same pathway in a similar direction; divergent, where treatments produced opposing or pathway-specific effects; or non-additive, where combined treatment effects could not be explained by the sum of individual effects. Attention is focused on non-linear results in the tonic group, as these results may reflect more complex network-wide effects rather than simple additive mechanisms.

### 5.5. Integrative Model Construction

Our analysis of UQ and ASH mechanistic interactions will be used to create an integrative schematic model which represents the prospective biochemical and cellular mechanisms of action. Pathway relationships are defined based on known biological interactions (e.g., oxidative stress regulating autophagy, glial responses influencing synaptic remodeling) and supported by other published observed marker patterns [61,63,64].

### 5.6. Probable Mechanistic Summaries of UQ + ASH

**Figure 1:**
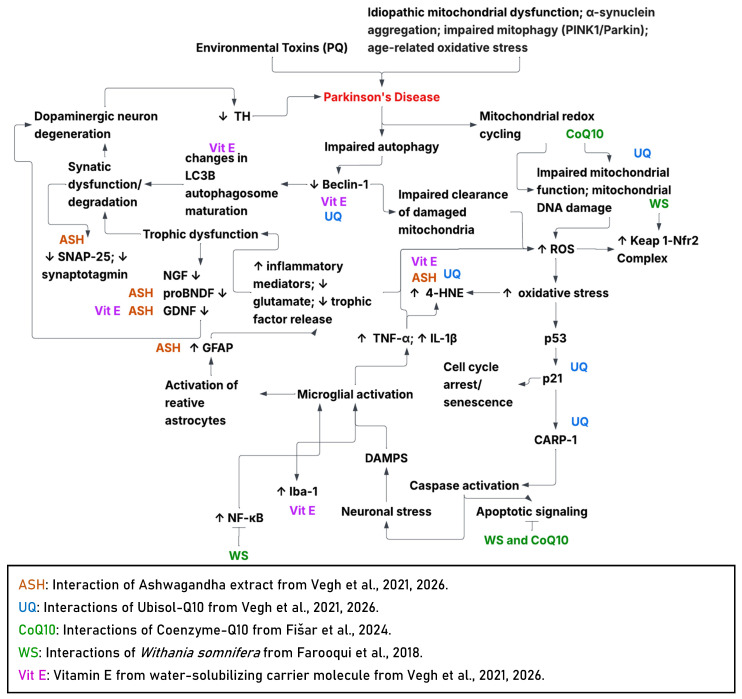
Integrative schematic illustrating the biochemical pathways underlying Parkinson’s disease (PD, written in red to highlight the disease being exemplified in the current figure) and the mechanistic points of action of the tonic treatment components (Ubisol-Q10 (UQ) and Ashwagandha (ASH)). ASH and UQ (shown in orange and blue respectively) represent findings of past published work from our lab [61,63] and indicate their reported points of influence within PD-related biochemical pathology. Coenzyne-Q10 (CoQ10) and *Withania somnifera* (WS), shown in green, represent findings from external studies [9,93]. Detailed statistical analysis should be consulted from the original publications. Up and down arrows next to each protein represent the disease-induced direction of expression typically seen for that marker.

**Figure 2:**
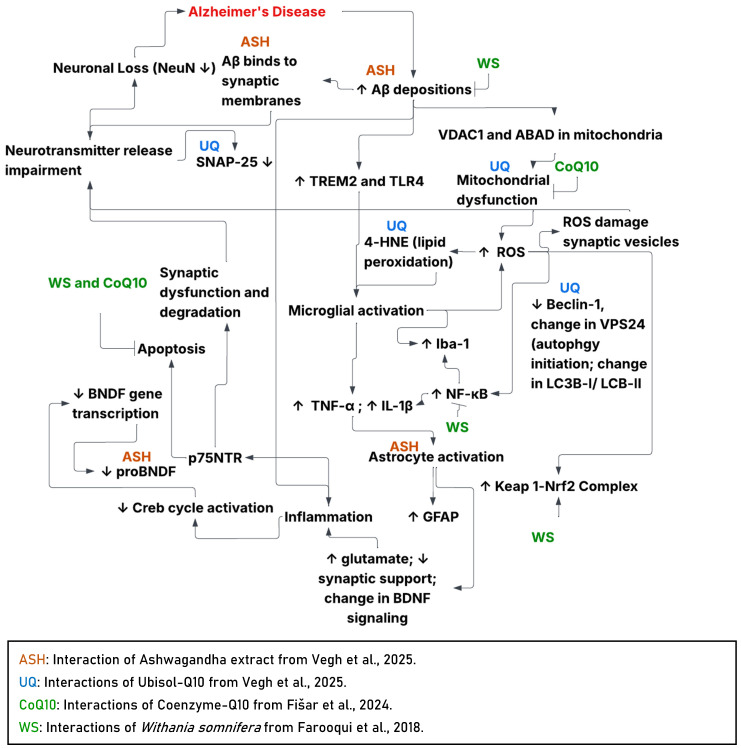
Integrative schematic illustrating the biochemical pathways underlying Alzheimer’s disease (AD, written in red to highlight the disease being exemplified in the current figure) and the mechanistic points of action of the tonic treatment components (Ubisol-Q10 (UQ) and Ashwagandha (ASH)). ASH and UQ (shown in orange and blue respectively) represent findings of past published work from our lab [64] and indicate their reported points of influence within AD-related biochemical pathology. Coenzyme-Q10 (CoQ10) and *Withania somnifera* (WS), shown in green, represent findings from external studies [9,93]. Detailed statistical analysis should be consulted from the original publications. Up and down arrows next to each protein represent the disease-induced direction of expression typically seen for that marker.

Figure 1 and Figure 2 display the probable mechanistic interactions of UQ/CoQ10, ASH, and vitamin E (from PTS) in AD and PD, as interpreted from Vegh et al., 2021, 2025 and 2026 [61,63,64]’s biochemical measures. Table 3 describes the outcomes of each paper which were used to propose the combined mechanistic interactions of UQ and ASH in Figure 1 and Figure 2.

## 6. Discussion of Possible Mechanisms of a Multi-Component Therapeutic Solution of UQ and ASH

The present study highlights distinct, yet complementary mechanisms of neuroprotection mediated by ASH and UQ, while also pinpointing important differences in treatment responses across disease models, and formulation conditions. These findings show that therapeutic efficacy of the tonic solution is highly effective across varying disease severity, stage of pathology, and formulation-related nutra-pharmacology.

Because CoQ10/UQ primarily acts as a mitochondrial electron transport chain stabilizer and antioxidant [94,95], its effects were more pronounced in the PQ-PD model, since the dominant motive of upstream disease progression is due to mitochondrial dysfunction. In contrast, ASH provided more prominent effects in the AD model, probably due to its well-known ability to enhance neurotrophic signaling, promote astrocytic repair, and modulate synaptic plasticity [73,96]. Moreover, the combined tonic treatment consistently produced the most balanced outcome across both AD and PD models. As per the schematic diagrams outlining the combined mechanistic effects of UQ and ASH (Figure 1 and Figure 2), it becomes apparent that UQ is mostly responsible for suppression of upstream oxidative damage while ASH simultaneously promotes downstream repair and neuronal remodeling. The tonic therefore appeared to work by directing disease pathology away from pro-inflammatory and pro-oxidative stress and providing a more controlled environment to enhance plasticity, rather than just suppressing the effects of damage as it occurs.

Interestingly, the PTS-ASH experiment [63] introduced two key variables: a lower ASH concentration (61 mg/kg/day) and a markedly increased concentration of the PTS carrier solution (20% increase in PTS amount provided in drinking solution), which contains vitamin E. Vitamin E is a membrane-localized antioxidant that primarily suppresses lipid peroxidation rather than mitochondrial ROS generation [97]. In PQ-injected rats treated with PTS-ASH, oxidative stress was shown to be relatively similar to E-ASH-treated rats; however, astrocyte activation was markedly reduced in the PTS-ASH group compared to the E-ASH. This is consistent with reports that vitamin E can suppress astrocytic calcium signaling and pro-inflammatory pathology via reduction in accumulation of lipid peroxidation products [98,99]. On a related note, autophagy markers were much more expressed in the PTS-ASH group which may be related to astrocytic control over the delivery of vitamin E to neurons, and their homeostatic response to oxidative stress [98]. The combination of reduced astrocyte activation and increased autophagy suggests that the increase in PTS carrier molecule may hold additional benefits in moderating glial-focused repair mechanisms and promoting proteostasis.

Nonetheless, several limitations should be considered. This analysis is based on the integration of previously published results and, therefore, does not include direct experimental validation of the inferred mechanistic interactions. Additionally, while widely used, the PQ-induced PD model and transgenic AD models do not fully recapitulate the sporadic and heterogeneous nature of human disease. The 5XFAD mouse model is based on genetic factors of familial AD, having inherent differences from sporadic AD [100]. Similarly, the PQ-induced PD model only reproduces oxidative stress and dopaminergic neuronal loss, lacking the chronic progressive nature of PD in humans and its respective symptoms [101]. Differences in species, disease progression timelines, and experimental design further limit direct translation of findings. As such, interpretations should focus on relative pathway dynamics rather than absolute equivalence to human pathology. To date, there is no preclinical model that recapitulates the full spectrum of AD or PD.

We would like to highlight here some important considerations that must be made before the combined UQ and ASH therapy can be considered clinically translatable for AD and PD. Dosing regimens effective in animal studies may not be directly scalable in humans, and the optimal therapeutic ratio of the two compounds remains undefined as per FDA standard. Furthermore, variability in ASH extract composition, including differences in withanolide content and extraction methods, presents challenges for formulation standardization and reproducibility across studies. While UQ improves the bioavailability of CoQ10, the pharmacokinetic profiles, blood–brain barrier penetration, and tissue distribution of both compounds in combination require further characterization outside of animal models. Long-term safety data are limited, particularly regarding chronic administration in older populations and potential herb–drug interactions with commonly prescribed medications. Additionally, Health Canada regulations for the combination of nutraceutical products remain less clearly defined than those for conventional pharmaceuticals, creating challenges for quality control, approval pathways, and clinical studies. Addressing these limitations will be essential to establish the efficacy, safety, and translational potential of this therapeutic approach. These points emphasize an important gap in medicinal translation of NHPs and may contribute to the limit of their perceived seriousness. Large-scale clinical trials of NHPs for treatment of neurodegenerative diseases should be considered at equal with those performed for pharmaceutical interventions and are necessary to develop safety parameters around administration.

Despite these limitations, the current review provides a comparative and generalizable integrative interpretation and prospective mechanistic model of neurodegeneration and co-administered therapeutic modulation. Future work integrating longitudinal validation, additional novel formulations, and translational studies will be essential for advancing these findings toward clinical application.

From a practical perspective, the results discussed in the current report support the development of combination-based therapeutic strategies that integrate mitochondrial protection, antioxidant defense, and neurotrophic repair. Such approaches may offer more robust and sustained benefits across heterogeneous neurodegenerative conditions compared to conventional monotherapies. Moreover, the integrative framework presented here provides a potentially valuable tool for identifying points of convergence and divergence across disease pathways, enabling more precise targeting of complex biological networks.

## 7. Conclusions and Future Perspectives

Here, we reviewed topics of various nutraceutical therapies for targeting AD and PD progression, their novel formulation strategies to enhance bioavailability, and co-administration of natural agents to provide more widespread pathological targeting. Combination therapies that integrate mitochondrial protection, antioxidant support, and trophic repair may provide the most all-inclusive outcomes across neurodegenerative conditions.

In addition, we sought to elaborate on previously published qualitative and semi-quantitative immunohistochemical evidence from our lab [61,63,64] to support the combination tonic therapy of UQ + ASH in AD and PD animal models. With this, we generated a prospective model of combined therapeutic impact across AD and PD mechanistic cascades to gain a more in-depth understanding of the combined benefits. Overall, the complementary mechanisms comprehensively discussed here suggest UQ stabilizes mitochondria and defends against oxidative damage, while ASH simultaneously promotes adaptive plasticity and glial-mediated repair. The addition of higher concentrations of vitamin E in the PTS-ASH formulation further improves membrane stability and autophagy. Nonetheless, the current review introduces a general mechanistic model, with which we hope to encourage advancement of validation of these proposed mechanisms through additional quantitative measures.

Together, we aim to elucidate the potential of combination therapies that integrate mitochondrial protection, antioxidant support, and trophic repair, to provide broader benefits across neurodegenerative conditions, while also maximizing the bioavailability and therapeutic efficacy of lipid-soluble neuroprotective agents using novel formulations. The preclinical evidence of these natural agents in combination and their novel formulations aim to support their future evolvement to clinical trials with humans.

## Figures and Tables

**Table 1:** Summary of published preclinical studies investigating the neuroprotective effects of Ubisol-Q10 (UQ), Ashwagandha (ASH), and combined tonic formulations in animal models of Alzheimer’s disease (AD) and Parkinson’s disease (PD) [61,63,64]. The table outlines the experimental animal models, treatment formulations and dosages, routes of administration, and treatment durations used across the included studies. UQ = Ubisol-Q10; ASH = Ashwagandha (*Withania somnifera*); PTS = polyoxyethanyl-α-tocopheryl sebacate-solubilizing molecule; b.w. = body weight; p.o. = per oral administration.

Source	Animal Model	Control or Treatment and Doses	Duration of Treatment
[64]	AD: Male B6-C3-Tg (APPswe/PSEN1dE9) mice Control: Male C57BL/6 wild-type mice	Experimental: 8 mg/kg/day b.w. p.o. UQ; 320 mg/kg/day b.w. p.o. ethanolic ASH extract; tonic of 8 mg/kg/day UQ + 320 mg/kg/day ethanolic ASH extract (b.w. p.o.) Control: AD and wild-type mice received water	Daily administration over 18 months
[61]	PD: Male Long-Evans hooded rats, paraquat-induced PD (10 mg/kg b.w. p.i.) Control: Male Long-Evans hooded rats, saline-injected	Experimental: 6 mg/kg/day b.w. p.o. UQ; 244 mg/kg/day b.w. p.o. ethanolic ASH extract; tonic of 6 mg/kg/day UQ + 122 mg/kg/day ethanolic ASH extract (b.w. p.o.) Note: experimental solutions were provided for both PD and healthy rat groups Control: PD and healthy rats received either water or water with 0.1% PTS	Daily administration over 4 months post-injection
[63]	PD: Male Long-Evans hooded rats, paraquat-induced PD (10 mg/kg b.w. p.i.) Control: Male Long-Evans hooded rats, saline-injected	Experimental: 6 mg/kg/day b.w. p.o. UQ; 61 mg/kg/day b.w. p.o. PTS-ASH; tonic of 6 mg/kg/day UQ + 61 mg/kg/day PTS-ASH (b.w. p.o.) Control: healthy rats received water and PD rats received water with 20% PTS	Daily administration over 10 weeks post-injection

**Table 2:** Integrative categorization of immunofluorescent biochemical and functional markers used in [61,63,64] to evaluate neurodegenerative disease progression and therapeutic responses in Alzheimer’s disease (AD) and Parkinson’s disease (PD) animal models. Biomarkers were organized into four progressive pathological domains: upstream pathological drivers, intracellular regulatory processes, cellular response systems, and functional outcomes.

Upstream Pathological Drivers	Intracellular Regulatory Processes	Cellular Response Systems	Functional Outcomes
Oxidative stress markers (4-hydroxynonenal (4-HNE)) Disease-specific proteotoxic burden (amyloid-β in AD)	Autophagy initiation and flux (Beclin-1, Microtubule-associated proteins 1A/1B light chain 3B (LC3B)) Cellular stress and senescence signaling (p21, assessed in PD only) Apoptosis (cell division cycle and apoptosis regulator 1 (CARP1), assessed in PD only)	Microglial activation (ionized calcium-binding adapter molecule 1 (Iba-1)) Astrocyte reactivity (glial fibrillary acidic protein (GFAP)) Neurotrophic signaling (nerve growth factor (NGF), assessed in PD only; glial-derived neurotrophic factor (GDNF), assessed in PD only; pro-brain-derived neurotrophic factor (pro-BDNF))	Synaptic function markers common to both diseases (synaptosomal-associated protein 25 kDa (SNAP-25)) PD-specific markers: dopaminergic neuronal integrity, assessed using tyrosine hydroxylase (TH); synaptic vesicle fusion, assessed using synaptotagmin PD-specific behavioral measure: motor performance using head up vs. down posture on a rotarod device; fine-motor coordination and gripping ability on a modified ladder apparatus containing an obstacle breakaway rung AD-specific markers: neuronal density and nuclear integrity assessed by neuronal nuclei (NeuN) AD-specific cognitive measure: novel object and novel location recognition test in an X-maze for evaluation of spatial and non-spatial working memory

**Table 3:** Summary of the qualitative neuroprotective and functional results using Ubisol-Q10 (UQ), Ashwagandha (ASH), and combined tonic formulations in preclinical animal models of Alzheimer’s disease (AD) and Parkinson’s disease (PD). All results summarized here have been previously published by our laboratory [61,63,64]. Detailed statistical analyses, including means, measures of variance, and significance testing, are available in the original publications cited. This table provides a qualitative summary of treatment outcomes across the three independent studies based on the immunofluorescent assays and behavioral/cognitive tests explained in Table 2. PTS = polyoxyethanyl-α-tocopheryl sebacate-solubilizing molecule; UQ = Ubisol-Q10, water-soluble formulation of coenzyme-Q10 with PTS; ASH = Ashwagandha (*Withania somnifera*); PTS-ASH = water-solubilized formulation of Ashwagandha with PTS.

Disease Animal Model	Solutions Administered	Biochemical Targets and Neuroprotective Outcomes	Tonic-Specific Highlights	Cognitive/Motor Behavioral Effects	Source
Male B6-C3-Tg (APPswe/PSEN1dE9) AD mice	Water control, UQ, ethanolic ASH, or tonic (UQ + ethanolic ASH extract)	Enhanced clearance of amyloid-β: UQ ~50%, ASH ~25%, tonic ~5% of transgenic untreated control expression of amyloid-β; Autophagy activation: UQ- and tonic-treated mice resulted near-equivalent to wild-type for markers of beclin-1 and microtubule-associated proteins 1A/1B light chain 3B (LC3B); Astrocytic support: ASH- and tonic-supplementation provided strong astrocyte activation in AD mice, resulting in ~5× higher expression of glial fibrillary acidic protein (GFAP) marker compared to untreated AD mice; Microglial inhibition: tonic-treated mice displayed the lowest expression of ionized calcium-binding adapter molecule 1 (Iba-1) at ~75% lower than wild-type and other treated groups, and >15× lower than untreated AD mice; Oxidative stress relief: tonic- and UQ-treated mice showed half the expression of 4-hydroxynonenal (4-HNE) compared to untreated AD mice; Trophic signaling balance: pro-brain-derived neurotrophic factor (pro-BDNF) expression was equivalent amongst wild-type, UQ- and tonic-treated groups, and ASH-treated AD mice showed ~2× higher expression than other treated groups, suggesting strong withanolide-driven repair signaling; Promotion of neuronal and synaptic plasticity: tonic-treated AD mice resulted in the highest expression of neuronal nuclei (NeuN) across all experimental and control groups, around double that of UQ- and ASH-treated AD mice, whose expression remained near-equivalent to wild-type; ASH- and tonic-treated AD mice expressed synaptosomal-associated protein 25 kDa (SNAP-25) at double the amount of untreated AD mice, and UQ-treated AD mice remained at-par with wild-type;	Tonic provided greatest reduction in plaque size/quantity and Iba-1 expression, highest neuronal nuclei (NeuN), pro-BDNF, and SNAP-25 expression	Tonic-treated and UQ-treated AD mice maintained long-term and working spatial memory equivalent to wild-type mice, and ASH-treated mice maintained 50% above untreated mice in the novel location recognition test; UQ-, ASH-, and tonic-treated AD mice maintained non-spatial memory at parity with wild-type mice in the novel object recognition test in an X-maze	[64]
Male Long-Evans hooded rats, paraquat (PQ)-induced PD (10 mg/kg b.w. p.i.)	Water control, PTS control, UQ, ethanolic ASH, or tonic (UQ + ethanolic ASH extract)	Activation of autophagy: tonic- and UQ-treated PQ-injected rats expressed beclin-1 at equivalent levels to healthy saline-injected rats, while ASH-treated PQ-injected rats showed expression at double that of untreated PD rats; PQ-injected rats treated with only PTS showed double the expression of the PQ-water control group, suggesting vitamin E even at low doses may play a role in autophagy resumption; Reduction in oxidative stress: PQ-injected rats treated with UQ and tonic resulted in ~100× reduction, and ASH-treated PQ-injected rats resulted in ~200× reduction in 4-HNE expression compared to PQ-injected untreated rats; interestingly, PQ-injected rats fed with PTS showed nearly double the expression for 4-HNE compared to their water-fed cohorts, suggesting vitamin E may play a role in modifying oxidative stress-related pathways; Inhibition of microgliosis: ASH-treated PD rats had the lowest reduction in expression of Iba-1 at ~50% of saline-injected healthy rats, and UQ-treated and tonic-treated PQ-injected rats showed expression ~75% of healthy control, which is half the expression from untreated OD rats; PQ-injected rats treated with PTS has ~2× higher expression of Iba-1 compared to water-fed cohorts, suggesting vitamin E may play a role in promoting microglial phagocytic pathways; Enhanced astrogliosis: ASH- and tonic-treated PD rats resulted in over 3× the GFAP expression of untreated PD rats, and UQ-treated PD rats showed 2× the expression; Upregulation of neurotrophic factors: ASH-treated PD rats expressed glial-derived neurotrophic factor (GDNF) and pro-BDNF at the highest amount compared to all other treatment and healthy control groups, and tonic-treated PD rats showed expression at ~3× higher than untreated PD rats; Cell death and apoptosis regulation: PQ-injected rats treated with UQ and tonic expressed cell division cycle and apoptosis regulator 1 (CARP1) near-equivalent to healthy saline-injected rats, and ASH-treated rats showed expression 300% higher than healthy control rats; Preservation of dopamanergic neurons: tyrosine hydroxylase (TH) expression in all treatment groups remained comparable to healthy saline-injected rats, whereas untreated PD rats showed very minimal expression (~5% of healthy control group)	DA neurons of tonic-treated PD-rats remained near-identical to healthy control (maintained number and morphology); stabilized tyrosine hydroxylase (TH); Tonic-treated saline-injected healthy rats showed interesting upregulation of GFAP, pro-BDNF (both ~2× higher than water-fed cohorts), and GDNF expression (~4× higher than water-fed healthy control group), suggesting tonic may support trophic signaling and plasticity via astrocyte-mediated pathways in healthy animals	Tonic-treated PD rats showed the strongest protection against motor impairment (least amount of time with head-down position on rotarod) compared to other treatment groups, and 2× better performance than untreated PD rats	[61]
Male Long-Evans hooded rats, paraquat-induced PD (10 mg/kg b.w. p.i.)	PTS control, UQ, PTS-ASH, or tonic (UQ + PTS-ASH)	Comprehensive targeting of oxidative stress: tonic-treated PD rats resulted in lowest 4-HNE expression (~15% lower than UQ-treated PD-rats and healthy control), and PTS-ASH-treated PD rats remained similar to the healthy control group; Resumption of autophagy: tonic-treated PD rats resulted in highest LC3B/beclin-1 expression, with beclin-1 expression ~5× higher and LC3B ~3× higher than untreated PD rats and 2× higher than healthy control, UQ-, and PTS-ASH-treated groups; PTS-ASH-treated PD rats resulted in 2× higher beclin-1 expression compared to UQ-treated and healthy control rats, which remained comparable; Improved cell cycle regulation: protein p21 expression was ~4× lower in PTS-ASH-treated PQ-injected rats, ~10× lower in UQ-treated PD rats, and ~5× lower in tonic-treated PD rats compared to unhealthy PQ-injected rats; Reduced inflammation and improved glial-focused byproduct clearance: UQ- and PTS-ASH-treated rats showed Iba-1 expression lower than even healthy control, and tonic-treated rats showed iba-1 expression at 800% of healthy control group, suggesting possible synergistic effects or complementary upstream targets upregulate glial-focused repair and debris; PTS-ASH- and tonic-treated PD rats expressed GFAP nearly 2× higher than untreated PD rats; Neurotrophic signaling balance: tonic-treated PD rats showed the highest expression of pro-BDNF compared to all experimental and control groups; however, all treated PD groups showed >2× higher expression compared to healthy control and >25× higher than the unhealthy PD control group; PTS-ASH- and UQ-treated PD rats remained comparable to healthy control rats in expression of GDNF; interestingly, tonic-treated PD rats showed GDNF expression about half that of the other treated groups, and much less than the expression found in the previous PD paper [61]. The same trend is found between the E-ASH results [61] vs. PTS-ASH results, suggesting the increased vitamin E may play a moderating role in GDNF pathways; all treated PD groups resulted in nerve growth factor (NGF) expression 2–3× higher than untreated PQ-injected rats; Improved synaptic and neuronal integrity: tonic-treated PD rats expressed the highest amount of SNAP-25 and synaptotagmin, ~10× higher than untreated PD rats, with PTS-ASH-treated rats slightly lower, and UQ-treated rats expressing SNAP-25 equivalent to healthy control and synaptotagmin 2× higher; TH expression remained comparable to healthy control rats across all treated PD groups, while PQ-injected untreated rats has >75% lower TH expression.	Tonic-treated PQ-injected rats showed maintained dopaminergic neuroprotection; maintained neuron count and fiber morphology; highest expression of synaptic markers, pro-BDNF, beclin-1 and LC3B; lowest expression of 4-HNE	Tonic-treated PD rats showed statistically significant increase in upward head posture (rotarod); maintained fine-motor gripping abilities (ladder test) comparable to healthy subjects	[63]

## Data Availability

No new data were created or analyzed in this study. Data sharing is not applicable to this article.

## Abbreviations

The following abbreviations are used in this manuscript:

ATP	Adenosine triphosphate
AD	Alzheimer’s disease
PD	Parkinson’s disease
NHPs	Natural health products
Aβ	Amyloid-β
ROS	Reactive oxygen species
DA	Dopaminergic
NMDA	*N*-methyl-*D*-aspartate
CUR	Curcumin
CoQ10	Coenzyme Q10
ETC	Electron transport chain
UQ	Ubisol-Q10
PTS	Polyoxyethanyl-α-tocopheryl sebacate
ASH	Ashwagandha
